# Potential Use of a Serpin from *Arabidopsis* for Pest Control

**DOI:** 10.1371/journal.pone.0020278

**Published:** 2011-05-31

**Authors:** Fernando Alvarez-Alfageme, Jafar Maharramov, Laura Carrillo, Steven Vandenabeele, Dominique Vercammen, Frank Van Breusegem, Guy Smagghe

**Affiliations:** 1 Laboratory of Agrozoology, Department of Crop Protection, Faculty of Bioscience Engineering, Ghent University, Gent, Belgium; 2 VIB Department of Plant Systems Biology, Ghent University, Gent, Belgium; 3 Department of Plant Biotechnology and Genetics, Ghent University, Gent, Belgium; Instituto de Biología Molecular y Celular de Plantas, Spain

## Abstract

Although genetically modified (GM) plants expressing toxins from *Bacillus thuringiensis* (*Bt*) protect agricultural crops against lepidopteran and coleopteran pests, field-evolved resistance to *Bt* toxins has been reported for populations of several lepidopteran species. Moreover, some important agricultural pests, like phloem-feeding insects, are not susceptible to *Bt* crops. Complementary pest control strategies are therefore necessary to assure that the benefits provided by those insect-resistant transgenic plants are not compromised and to target those pests that are not susceptible. Experimental GM plants producing plant protease inhibitors have been shown to confer resistance against a wide range of agricultural pests. In this study we assessed the potential of AtSerpin1, a serpin from *Arabidopsis thaliana* (L). Heynh., for pest control. *In vitro* assays were conducted with a wide range of pests that rely mainly on either serine or cysteine proteases for digestion and also with three non-target organisms occurring in agricultural crops. AtSerpin1 inhibited proteases from all pest and non-target species assayed. Subsequently, the cotton leafworm *Spodoptera littoralis* Boisduval and the pea aphid *Acyrthosiphon pisum* (Harris) were fed on artificial diets containing AtSerpin1, and *S. littoralis* was also fed on transgenic *Arabidopsis* plants overproducing AtSerpin1. AtSerpin1 supplied in the artificial diet or by transgenic plants reduced the growth of *S. littoralis* larvae by 65% and 38%, respectively, relative to controls. Nymphs of *A. pisum* exposed to diets containing AtSerpin1 suffered high mortality levels (LC_50_ = 637 µg ml^−1^). The results indicate that AtSerpin1 is a good candidate for exploitation in pest control.

## Introduction

Herbivorous pests of major crops are estimated to reduce yields by 8–15% worldwide [1]. Engineering crop plants for endogenous resistance to insect pests has been an important success of molecular technology. Currently, genetically modified (GM) plants expressing δ-endotoxins from *Bacillus thuringiensis* (*Bt*) are providing significant control of agricultural insect pests and have reduced pesticide usage and production costs [2], [3]. The area sown with *Bt* crops has increased each year since 1996, when the first *Bt* crops were cultivated; in 2010, *Bt* crops were planted on 58 million hectares [4].

As farmers increasingly plant insect-resistant GM crops, selection pressure for the development of insect pests resistant to *Bt* toxins is also increasing. To date, field-evolved resistance has been documented in populations of five lepidopteran species [5]. Moreover, the efficacy of commercial *Bt* crops for some lepidopteran pests, such as the cotton leafworm *Spodoptera littoralis* Boisduval, is limited [6],[7], and phloem feeding pests including aphids are not susceptible to *Bt* crops [8]. Hence, complementary pest control strategies are necessary both to assure that the benefits provided by insect-resistant transgenic plants are not compromised and to target those pests that are not susceptible to *Bt* toxins. A summary of the strategies currently being investigated can be found in [8]–[11]. Among these, GM crops producing plant serine or cysteine protease inhibitors have been shown to confer resistance against a wide range of agricultural pests [12]. Protease inhibitors contribute to plant defense by inhibiting invertebrate proteases and, consequently, by reducing the availability of amino acids necessary for invertebrate growth and development. Transgenic plants expressing protease inhibitors, however, rarely achieve the same level of pest control as transgenic plants expressing *Bt* toxins [13] because herbivores are able to use several strategies to adapt to the inhibitors [12]. Still, plant protease inhibitors have the potential to be effective insecticidal proteins if insect adaptation to them can be overcome. For example, the combination of two protease inhibitors can lead to adverse effects on the target species that are not obtained with either inhibitor alone [14].

Serpins (serine protease inhibitors or classified inhibitor family I4) are the largest and most broadly distributed superfamily of protease inhibitors [15]. Serpin-like genes have been identified in animals, plants, bacteria, and some viruses [16]. Most serpins are irreversible inhibitors of serine proteases of the chymotrypsin family, although some have evolved to inhibit other types of serine proteases, and a few are also able to inhibit cysteine proteases [17]–[21]. Furthermore, some serpins have the ability to form complexes with very divergent proteases [22]. Serpins are involved in a number of fundamental biological processes, and a role in the protection of storage tissue against insects and pathogens has been proposed for plant serpins [23], [24]. Consistent with the idea that serpins protect against plant pests, the survival and fecundity of the green peach aphid *Myzus persicae* (Sulz.) were strongly and negatively correlated with the level of the serpin CmPS-1 in the phloem sap of *Cucurbita maxima* Duchesne [25]. A related serpin from *Cucurbita sativa* L., CsPS-1, is also thought to play a role in defense against herbivores [26].

Here we assessed the potential of AtSerpin1, a serpin from *Arabidopsis thaliana* (L). Heynh., for pest control. *In vitro* assays were conducted to measure the inhibitory activity of AtSerpin1 against a range of pest species that rely mainly on either serine or cysteine proteases for digestion. Because insect-resistant GM plants should ideally control target species without harming non-target arthropods, a decomposer, a pollinator, and a predator were included in these *in vitro* assays. Subsequently, two pest species, *S. littoralis* and the pea aphid *Acyrthosiphon pisum* (Harris), were used in *in vivo* assays on artificial diets containing AtSerpin1. Finally, transgenic *Arabidopsis* plants overproducing AtSerpin1 were tested against *S. littoralis*.

## Materials and Methods

### Invertebrates

#### Pest species

A permanent colony of *A. pisum* was reared on broad bean, *Vicia faba* L., plants. A laboratory colony of *S. littoralis* was maintained on an agar-based artificial diet. The two-spotted spider mite, *Tetranychus urticae* Koch, was reared on *Phaseolus vulgaris* L. bean plants in the laboratory, and the Colorado potato beetle, *Leptinotarsa decemlineata* (Say), was reared on fresh leaves of *Solanum tuberosum* L. in the laboratory. Frozen larvae of the Mediterranean corn borer, *Sesamia nonagrioides* Lefèbvre, were provided by Dr. Félix Ortego (Centro de Investigaciones Biológicas, CSIC, Madrid, Spain). All stages of a permanent insect colony of the red flour beetle, *Tribolium castaneum* Herbst, and of the yellow mealworm, *Tenebrio molitor* L., were kept on wheat flour mixed with brewer's yeast (10/1, w/w).

#### Non-target species

Large earth bumblebees, *Bombus terrestris* (L.), and green lacewings, *Chrysoperla carnea* (Stephens), were purchased from Biobest NV (Westerlo, Belgium) and reared in our laboratory for several generations with commercial sugar water and pollen and eggs of the flour moth *Ephestia kuehniella* Zeller, respectively. Common earthworms, *Lumbricus terrestris* L., were collected in an agricultural field in Ghent (Belgium) and frozen in the laboratory upon arrival.

All laboratory colonies were reared in environmental chambers at 24±2°C, 65±5% RH, and a 16∶8 h (L∶D) photoperiod.

### Preparation of extracts

Adults of *A. pisum*, *T. castaneum*, and *T. molitor*, and a mixture of all stages of *T. urticae* were collected from the rearing colonies, homogenized in 0.15 M NaCl, centrifuged at 10,000 *g* for 5 min and stored frozen at −20°C until needed. Last instar larvae of *S. littoralis*, *S. nonagrioides*, *L. decemlineata*, and *C. carnea*, and adults of *L. terrestris*, *B. terrestris*, and *C. carnea* were dissected in ice-cold 0.15 M NaCl, and the midguts and contents were removed. Each midgut was subsequently homogenized in 500 µl of 0.15 M NaCl. The suspensions were then centrifuged at 10,000 *g* for 5 min, and the supernatants were stored frozen at −20°C until needed. Before extracts were frozen, total protein content was determined according to the method of Bradford [27] with bovine serum albumin (BSA) as a standard.

### Production of recombinant AtSerpin1

Recombinant Atserpin1 was produced and purified as described in Vercammen et al. [33]. The cDNA for the ORF of At1g47710 was obtained by RT-PCR with the following forward and reverse primers, provided with the adequate 5′ extensions by Gateway® cloning (Invitrogen, Merelbeke, Belgium): 5′-ATGGACGTGCGTGAATC-3′ and 5′-TTAATGCAACGGATCAACAAC-3′. After recombination in pDEST17, the plasmid was introduced into *E. coli* strain BL21(DE3)pLysE, and production of the HIS6-tagged protein was induced by incubation in 0.2 mM isopropyl-β-D-thiogalactopyranoside for 24 h. The protein was purified by metal ion affinity chromatography (TALO™; BD, Franklin Lakes, NJ). Protein concentration and purity were checked by Bradford analysis (Bio-Rad, Nazareth, Belgium) and SDS-polyacrylamide gel electrophoresis (PAGE).

### *In vitro* inhibitory activity of AtSerpin1 against invertebrate digestive proteases

To elucidate the potential of AtSerpin1 to inhibit invertebrate digestive proteases, several species known to rely either on serine or cysteine proteases for protein digestion were selected for *in vitro* experiments. Specifically, the ability of AtSerpin1 to inhibit the trypsin- and chymotrypsin-like serine activities in extracts of *S. littoralis*, *S. nonagrioides*, *T. molitor*, *L. terrestris*, *B. terrestris*, and *C. carnea* was tested using the substrates ZPR-AMC (*N*-carbobenzoxy-Phe-Arg-7-amido-4-methylcoumarin) and SLLVT-AMC (*N*-Suc-Leu-Leu-Val-Tyr-7-amido-4-methylcoumarin), respectively, and 0.1 M Tris-HCl buffer (pH 9.0). Inhibition of the cathepsin B- and L-like cysteine activities by AtSerpin1 in extracts of *A. pisum*, *T. castaneum*, *L. decemlineata*, and *T. urticae* was determined using the substrates ZRR-AMC (*N*-carbobenzoxy-Arg-Arg-7-amido-4-methylcoumarin) and ZPR-AMC, respectively, and 0.1 M phosphate buffer (pH 5.0). The standard assay used 5 µg of protein extract in a volume of 100 µl. AtSerpin1 was added at different final concentrations, ranging from 0.15 to 10 µM, and was incubated with the extracts for 15 min at room temperature. The substrate was then added to a final concentration of 0.2 mM. The reaction was incubated at 30°C for 45 min, and the emitted fluorescence was measured with a 365 nm excitation wavelength filter and a 465 nm emission wavelength filter. Results were expressed as a percentage of protease activity relative to that in the absence of the inhibitor. All assays were carried out in duplicate with pooled extracts.

### Generation of *Arabidopsis* plants overproducing AtSerpin1

Transgenic *Arabidopsis* plants overproducing AtSerpin1 were generated to further investigate the potential of the serpin against *S. littoralis* larvae. The cDNA for the ORF of At1g47710 was obtained by reverse transcription-PCR with the following forward and reverse primers, provided with the adequate 5′ extensions for Gateway® cloning: 5′-ATGGACGTGCGTGAATC-3′ and 5′-TTAATGCAACGGATCAACAAC-3′. The ORF was cloned into the binary vector pB7GW2 [28] via Gateway® recombination. In the resulting vector, the ORF was under transcriptional control of the promoter of the cauliflower mosaic virus 35S (CaMV 35S); the glufosinate ammonium resistance gene was present to allow for transgene selection. Binary constructs were transformed into *Agrobacterium tumefaciens* strain C58C1RifR[pMP90], and transgenic *Arabidopsis* Columbia-0 were obtained via floral dip transformation [29] and subsequent selection. Serpin overexpression was assessed by immunoblotting using antisera against AtSerpin1 [19]. Three single-locus homozygous lines with high transgenic protein expression were selected for further analysis by Western blot.

### *In vivo* effect of AtSerpin1

*In vivo* experiments with *S. littoralis* and *A. pisum* were used to assess the potential of AtSerpin1 for pest control. These two herbivorous species were selected because (i) they are serious pests of several agricultural crops, (ii) they rely on different proteolytical enzymes for protein digestion, and (iii) our *in vitro* studies demonstrated that they are both highly susceptible to AtSerpin1 (see Results).

#### Effect of purified AtSerpin1 on *S. littoralis*

Third-instar *S. littoralis* larvae (8–10 mg each) from the laboratory colony were starved for 4 h before the start of the bioassay. Subsequently, four larvae were placed in a Petri dish (9 cm diameter) and fed *ad libitum* for 6 days with artificial diet containing 0 (control), 65, or 650 µg g^−1^ AtSerpin1. Larvae were weighed on day 2, 4, and 6. Each treatment was represented by 12 replicate Petri dishes.

At the end of the feeding assay, 24 larvae from the control and the 65 µg g^−1^ AtSerpin1 treatment were selected randomly and the midguts were dissected. Susbsequently, the serine-like proteolytic activites trypsin, chymotrypsin, and elastase were quantified as described by Ortego et al. [30].

#### Effect of transgenic *Arabidopsis* overproducing AtSerpin1 on *S. littoralis*

Second-instar *S. littoralis* larvae (2.5–3.0 mg each) from the laboratory colony were starved for 4 h and transferred to pots planted with 4-week-old *Arabidopsis*: transgenic lines overproducing AtSerpin1 (lines AtSerpin^OE1^, AtSerpin^OE2^, and AtSerpin^OE3^) or the non-transformed line Col-0. Four larvae were confined per pot and allowed to feed for 4 days. Larvae were weighed on the second and the fourth day. Six pots per line were used, resulting in 24 larvae per treatment.

Both bioassays with *S. littoralis* were carried out in a growth chamber at 24±2°C, 65±5% RH, and a 16∶8 h (L∶D) photoperiod.

#### Effect of purified AtSerpin1 on *A. pisum* survival

Reproductive adults from the *A. pisum* laboratory colony were collected and transferred to fresh bean leaves, where they were allowed to produce nymphs for 12 h. Experimental arenas consisted of sachets containing 130 µl of artificial diet as described by Shahnaz et al. [31]. Neonate (<12 h) *A. pisum* nymphs were then brushed carefully onto sachets containing AtSerpin1 at concentrations ranging from 0 to 1000 µg ml^−1^. Fifteen nymphs were confined in each sachet, and three to six sachets were used per treatment. Nymphal survival was recorded after 3 days, and Abbott's correction for natural mortality was applied [32].

#### Effect of purified AtSerpin1 on *A. pisum* proteolytic activities

Neonate nymphs (<12 h) from the permanent *A. pisum* culture were placed on sachets containing 0 or 1000 µg ml^−1^ AtSerpin1, and allowed to feed for 24 h. Three sachets containing 15 nymphs were used per treatment. After the feeding period, aphids from every sachet were collected, homogenized in 0.15 M NaCl, and stored frozen at −20°C until required. Finally, digestive enzyme activities were measured as described by Carrillo et al. [33].

Both bioassays with *A. pisum* were performed in a growth chamber at 24±2°C, 65±5% RH, and a 16∶8 h (L∶D) photoperiod.

### Statistical analysis

A one-way analysis of variance (ANOVA) followed by a Student-Newman-Keuls test was used to compare *S. littoralis* larval growth among the different treatments in both bioassays and to compare the proteolytic activities of *A. pisum* fed with artificial diet with or without AtSerpin1. Proteolytic activities of *S. littoralis* larvae fed either with control diet or diet incorporating AtSerpin1 were analyzed using the Mann-Whitney *U* test because data were not normally distributed. Differences between treatments were considered significant at *P*<0.05. The concentration of AtSerpin1 causing 50% mortality (LC_50_) on aphid nymphs was analyzed using nonlinear sigmoid curve fitting using the GraphPad Prism 4.0 software (GraphPad Software Inc., La Jolla, CA).

## Results

### *In vitro* inhibitory activity of AtSerpin1 against invertebrate digestive proteases

The inhibitory activity of AtSerpin1 was tested *in vitro* against serine or cysteine proteases from several invertebrate pest and non-target species (Table 1). The inhibition of trypsin- and chymotrypsin-like serine activities was investigated in extracts of the pests *S. littoralis*, *S. nonagrioides*, and *T. molitor*, and in extracts of the non-targets *L. terrestris*, *B. terrestris*, and *C. carnea* (Table 2). For all species, inhibition of trypsin activity by 10 µM AtSerpin1 was higher than 80%. The trypsin activities of the non-targets *C. carnea* larvae and *B. terrestris* were highly susceptible to AtSerpin1, with an inhibition of 70% and 90%, respectively, at the lowest concentration tested (0.15 µM). AtSerpin1 also inhibited chymotrypsin activity in all species tested, except in the case of *S. nonagrioides*.

**Table 1:** Ecological function and main digestive proteases of the invertebrate species tested *in vitro* against AtSerpin1.

Species name	Ecological function	Main proteases	Reference
*Spodoptera littoralis* (Lepidoptera: Noctuidae)	Herbivory	SEP	[34]
*Sesamia nonagrioides* (Lepidoptera: Noctuidae)	Herbivory	SEP	[30]
*Tenebrio molitor* (Coleoptera: Tenebrionidae)	Herbivory	SEP, CEP*	[35]
*Lumbricus terrestris* (Annelida: Lumbricidae)	Decomposition	SEP	[36]
*Bombus terrestris* (Hymenoptera: Apidae)	Pollination	SEP	[37]
*Chrysoperla carnea* (Neuroptera: Chrysopidae)	Predation	SEP	[38]
*Acyrthosiphon pisum* (Homoptera: Aphididae)	Herbivory	CEP	33
*Tribolium castaneum* (Coleoptera: Tenebrionidae)	Herbivory	CEP	[39]
*Leptinotarsa decemlineata* (Coleoptera: Chrysomelidae)	Herbivory	CEP	[40]
*Tetranychus urticae* (Acari: Tetranychidae)	Herbivory	CEP	[41]

Abbreviations: SEP = serine endoproteases; CEP = cysteine endoproteases.

*Only SEP were tested against AtSerpin1.

**Table 2:** *In vitro* inhibitory activity of the protease inhibitor AtSerpin1 against trypsin- and chymotrypsin-like serine, and cathepsin B- and cathepsin L-like cysteine activities in extracts of several pest and non-target invertebrate species.

	Inhibition (%)					
	Trypsin activity			Chymotrypsin activity		
Species name	0.15 µM	1.25 µM	10 µM	0.15 µM	1.25 µM	10 µM
*Spodoptera littoralis* *	11.8±0.8	52.3±0.3	90.4±0.7	18.2±4.0	30.7±15.3	39.7±14.8
*Sesamia nonagrioides* *	36.0±1.3	89.8±1.3	97.5±0.3	ni	ni	ni
*Tenebrio molitor* *	51.2±3.5	94.9±0.6	92.3±3.6	7.2±0.8	14.1±2.3	45.3±3.8
*Lumbricus terrestris* †	ni	ni	82.7±1.2	-	-	-
*Bombus terrestris* †	91.8±0.5	97.8±0.3	98.5±0.1	-	-	-
*Chrysoperla carnea* †						
Larvae	66.6±3.5	94.0±3.2	98.8±1.3	ni	51.1±7.2	75.5±1.3
Adults	56.2±1.5	99.2±0.4	99.5±1.0	ni	15.8±2.5	66.1±5.7

	Cathepsin B activity			Cathepsin L activity		
	0.15 µM	1.25 µM	10 µM	0.15 µM	1.25 µM	10 µM
*Acyrthosiphon pisum* *	38.6±1.3	49.9±0.1	55.5±1.7	38.3±2.2	42.4±2.7	39.2±4.3
*Tribolium castaneum* *	ni	ni	75.1±3.6	ni	ni	69.8±3.5
*Leptinotarsa decemlineata* *	13.5±13.4	40.6±8.5	45.8±10.6	ni	3.9±9.9	47.8±3.8
*Tetranychus urticae* *	ni	ni	46.7±25.0	ni	ni	24.6±3.1

The percentage of inhibition was calculated as [(1 – activity with AtSerpin1/activity without AtSerpin1)×100]. Values represent mean+SE for duplicated independent determinations from a unique pool of extracts.

*pest species;

† non-target species.

“ni” denotes no inhibition.

The inhibition of cathepsin B- and L-like cysteine activities was determined in extracts of the pest species *A. pisum*, *T. castaneum*, *L. decemlineata*, and *T. urticae* (Table 2). AtSerpin1 inhibited the hydrolysis of the substrate ZRR-AMC in all species studied, although it never caused more than 75% inhibition, suggesting that cathepsin B activity is much less susceptible than trypsin activity to AtSerpin1. Inhibition of cathepsin L activity was also detected in these four species.

### Effect of AtSerpin1 on *S. littoralis*

#### Bioassay with artificial diet

**Figure 1:**
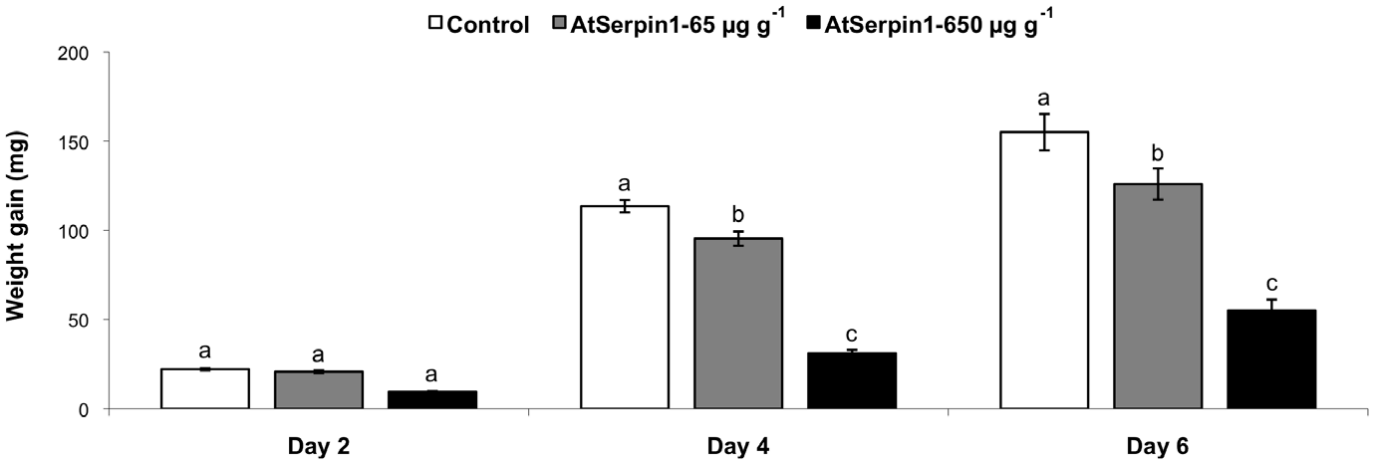
Weight gain of *Spodoptera littoralis* larvae fed on a diet containing 65 or 650 µg g^−1^ AtSerpin1 or control diet without inhibitor. Feeding assays were performed for 6 days with third-instar larvae. Bars represent mean ± SE. Bars with different letters on the same day are significantly different (*P*<0.05; one-way ANOVA followed by Student-Newman-Keuls) (*N* = 48).

**Figure 2:**
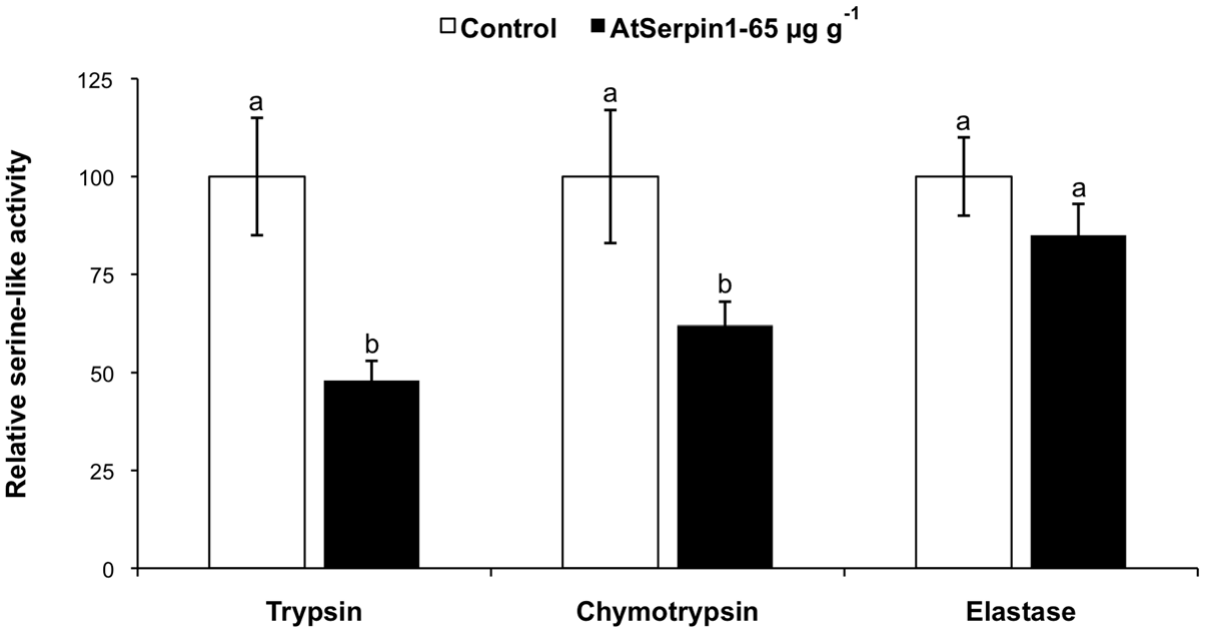
Serine-like proteolytic activities of *Spodoptera littoralis* third-instar larvae fed for 6 days on a diet containin 65 µg g^−1^ AtSerpin1 or control diet without inhibitor. Bars represent mean ± SE. Bars with different letters are significantly different (*P*<0.05; Mann-Whitney *U* test) (*N* = 24).

Ingestion of artificial diets containing the protease inhibitor AtSerpin1 markedly reduced the weight gain of *S. littoralis* (Figure 1). A significant difference (*P*<0.001) occurred after only 2 days of exposure when third instars were reared on artificial diet containing 650 µg g^−1^ AtSerpin1. This difference continued throughout the bioassay, and on day 6, the weight increase was 65% (*P*<0.001) lower for *S. littoralis* larvae ingesting the inhibitor than for the control. For larvae exposed to 65 µg g^−1^ AtSerpin1, weight gain was significantly reduced by 20% on day 4 and by 33% on day 6 relative to the control (Figure 2).

To investigate the physiological background, biochemical analysis were carried out on guts of *S. littoralis* larvae dissected at the end of the feeding assay. Trypsin and chymotrypsin activities were significantly reduced in those fed on artificial diet incorporating 65 µg g^−1^ AtSerpin1 compared to those feeding on control diet, whereas no differences were observed for elastase activity (Figure 2).

#### Bioassay with transgenic *Arabidopsis*

**Figure 3:**
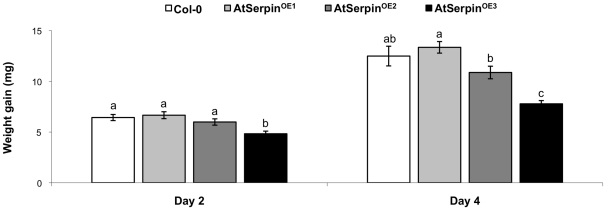
Western blot immunoassay showing the expression of AtSerpin1 in leaves of the transgenic *Arabidopsis* lines AtSerpin1^OE1^, AtSerpin1^OE2^, and AtSerpin1^OE3^, and the non-transformed line Col-0. Lanes: (1) Page ruler plus protein standard; (2) 100 ng AtSerpin1; (3) 50 ng AtSerpin1; (4) 25 ng AtSerpin1; (5) 12.5 ng AtSerpin1; (6) 5 ng AtSerpin1; (7) 0 ng AtSerpin1; (8) overproducing line AtSerpin^OE3^ (6 ng); (9) overproducing line AtSerpin^OE2^ (6 ng); (10) overproducing line AtSerpin^OE1^ (6 ng); (11) non-transformed line Col-0. In lanes 7–9, the upper band is the full-length and active form of AtSerpin1, while the lower band is the cleaved form after interaction with a protease.

Three transgenic *Arabidopsis* lines overproducing AtSerpin1 were tested against *S. littoralis* Expression of AtSerpin1 in leaves of *Arabidopsis* was confirmed by Western blot using increasing concentrations of purified AtSerpin1 (Figure 3). Differences in the AtSerpin1 expression among the transgenic lines was observed, being higher in AtSerpin^OE2^ and AtSerpin1^OE3^. In those lines, about 5 ng AtSerpin ug^−1^ of total protein content was measured.

**Figure 4:**
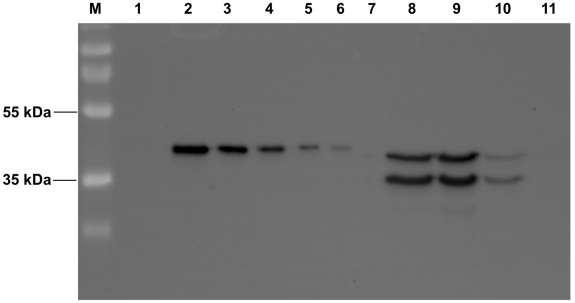
Weight gain of *Spodoptera littoralis* larvae fed on transgenic *Arabidopsis* plants overproducing AtSerpin1 (lines AtSerpin^OE1^, AtSerpin^OE2^, and AtSerpin^OE3^) or on non-transformed plants (line Col-0). Feeding assays were performed for 4 days with second-instar larvae. Bars represent mean ± SE. Bars with different letters on the same day are significantly different (*P*<0.05; one-way ANOVA followed by Student-Newman-Keuls) (*N* = 24).

Second-instar larvae were fed for 4 days on transgenic or non-transformed plants, and the increase of weight was measured (Figure 4). No significant differences were observed when the transgenic lines AtSerpin^OE1^and AtSerpin^OE2^ were compared with the control plants. However, the increase of weight was 25% lower (*P*<0.001) on day 2 and 38% lower (*P*<0.001) on day 4 for *S. littoralis* larvae reared on the transgenic AtSerpin^OE3^ than for larvae fed on non-transformed plants.

### Effect of AtSerpin1 on *A. pisum*

**Figure 5:**
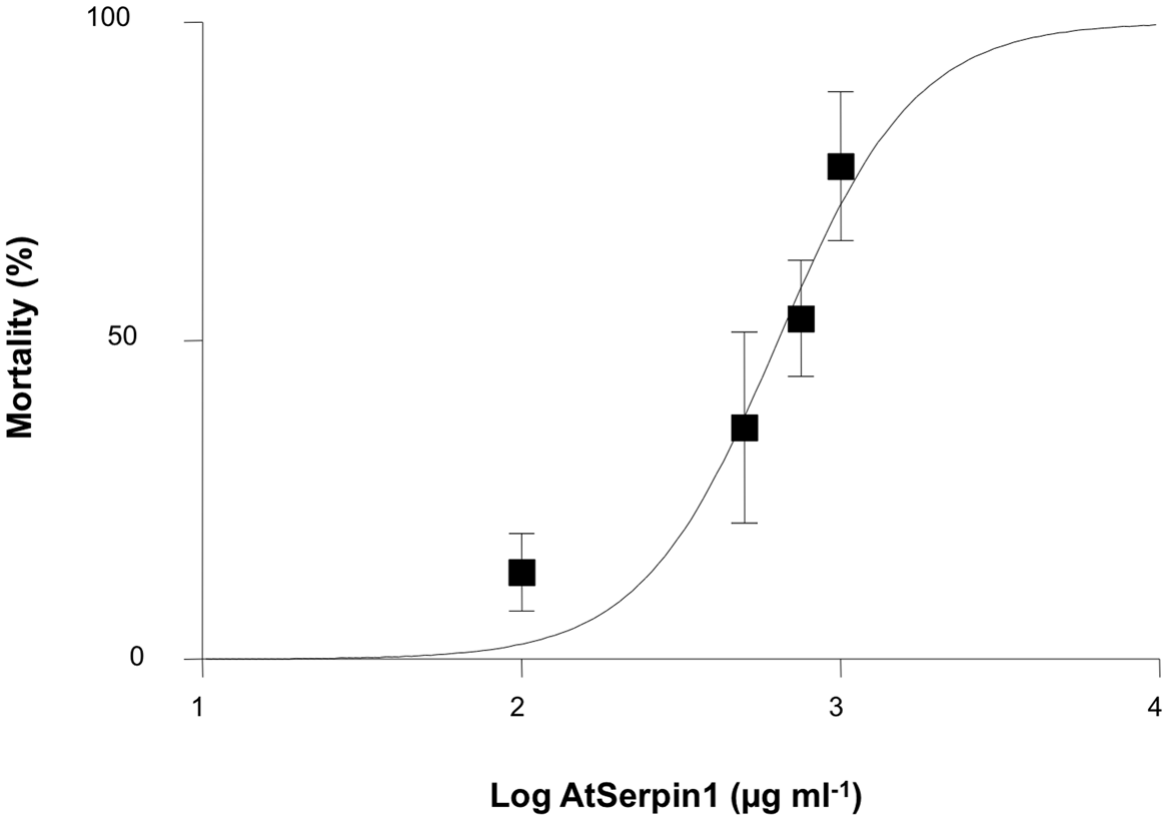
Concentration-response curve for mortality of newborn *Acyrthosiphon pisum* nymphs fed for 3 days with artificial diet containing increasing concentrations of the protease inhibitor AtSerpin1. Points represent mean ± SE. Three to six replicates with 15 nymphs each were used per concentration.

*A. pisum* nymphs reared for 3 days on diets containing 100 to 1000 µg ml^−1^ AtSerpin1 were highly susceptible to the inhibitor (Figure 5). Mortality reached 77.4% when *A. pisum* were fed 1000 µg ml^−1^ of the serpin. The effective AtSerpin1 concentration for 50% mortality (LC_50_) at the third day of feeding was 637 µg ml^−1^ (95% confidence limits = 367–1105; *R* ^2^ = 0.91) (Figure 5). Hence, it appears that AtSerpin1 not only inhibits cysteine proteases in *A. pisum* extracts *in vitro* but also has a strong insecticidal effect on nymphs.

To investigate the response of proteolytical enzymes of *A. pisum* to the ingestion of AtSerpin1, nymphs were fed with a diet containing 1000 µg ml^−1^ AtSerpin1 or a control diet without the inhibitor for 24 h, and proteolytic activities were subsequently quantified (Table 3). The cathepsin B- and L-like cysteine activities were significantly reduced (by 37% and 47%, respectively) in nymphs fed with AtSerpin1. In contrast, leucine aminopeptidase activity was enhanced by 42% when aphids were exposed to the inhibitor. Lastly, no differences were observed in carboxypeptidase A and B activities in *A. pisum* nymphs that were fed a diet with or without AtSerpin1.

**Table 3:** Proteolytic activities of *Acyrthosiphon pisum* adults after 1 day of feeding on a control diet (AtSerpin1−) or a diet containing 1000 µg ml^−1^ AtSerpin1 (AtSerpin1+).

		Specific activity^a^	
Protease	pH	AtSerpin1−	AtSerpin1+
Cysteine protease			
Cathepsin B	6.5	4.0±0.36^a^	2.5±0.19^b^
Cathepsin L	3	18.3±0.87^a^	9.6±0.34^b^
Cathepsin L	5.5	13.9±1.43^a^	7.8±0.47^b^
Leucine amino peptidase	7	8.7±0.93^a^	12.4±0.84^b^
Carboxypeptidase A	7	9.2±0.50^a^	8.4±0.40^a^
Carboxypeptidase B	8	12.6±0.44^a^	12.3±0.90^a^

a Specific activities as nmoles of substrate hydrolyzed min^−1^ mg protein^−1^. Values are mean ± SE of triplicate measurements from three independent replicates.

Means followed by the same letter within a row are not significantly different from each other (*P*≤0.05; one-way ANOVA followed by Student-Newman-Keuls).

## Discussion

Although many plant protease inhibitors from the serpin superfamily have been identified and hypothesized to have a role in host defense, to our knowledge only Yoo et al. [25] and the current study have investigated the potential of a serpin for pest control.

### *In vitro* inhibitory activity of AtSerpin1 against invertebrate digestive proteases

*In vitro* studies revealed that AtSerpin1 has a broad spectrum of activity because it inhibited both serine and cysteine proteases from a wide range of organisms, including the common earthworm (*L. terrestris*), the two-spotted spider mite (*T. urticae*), and eight insect species belonging to five different orders. Two recent studies have demonstrated the ability of AtSerpin1 to inhibit cysteine proteases [19], [42]; the potential of AtSerpin1 to target serine proteases, however, has never been reported before. Although most serpins inhibit either serine or cysteine proteases [16], some can inhibit proteases from several families. For example, the mouse serpin SON-5 is a dual inhibitor of both chymotrypsin-like serine and the papain-like cysteine proteases [43]. Brüning et al. [44] showed that a serpin, Spn4, from the fruit fly *Drosophila melanogaster* Meigen inhibits proteases from three different families.

The role of plant serpins in the protection of crops against insects has been proposed [23], [24], but very little is known about the potential of such protease inhibitors to control agricultural pests. To address this question, we selected two species, *S. littoralis* and *A. pisum*, for further *in vivo* studies (discussed in the next two sections). We selected these species in part because *S. littoralis* relies mainly on serine proteases, *A. pisum* relies mainly on cysteine proteases, and both were susceptible to AtSerpin1 in the *in vitro* experiments.

### *In vivo* effect of AtSerpin1 on *S. littoralis*

Serine proteases provide the major midgut endoproteolytic activities in *S. littoralis* larvae [34], and previous studies have demonstrated that transgenic plants expressing serine protease inhibitors can confer resistance against *S. littoralis* [45], [46]. When the protease inhibitor AtSerpin1 was incorporated into an artificial diet, the weight gain of *S. littoralis* larvae was substantially reduced relative to the control. The observed effects of AtSerpin1 on larval weight gain were correlated with a significant decreased of midgut trypsin activity. Weight gain also was reduced when *S. littoralis* were fed with transgenic *Arabidopsis* plants overproducing the serpin.

The results obtained in our bioassays are in agreement with many studies that have shown the potential of different plant serine protease inhibitors to interfere with the performance of lepidopteran species, either when the inhibitors are incorporated into an artificial diet or when they are expressed in transgenic plants [8], [12], [47]. However, the level of pest control that is routinely provided by *Bt* toxins is rarely provided by serine protease inhibitors, including AtSerpin1. It is well known that lepidopteran pests possess a remarkable ability to adapt their digestive proteolytic metabolism to the dietary material ingested and, therefore, to counteract the inhibitory activity of protease inhibitors [48], [49]. For this reason, researchers have suggested that a combination of two or more inhibitors may be required to overcome the capacity of such species to adapt to protease inhibitors. For example, Dunse et al. [14] recently demonstrated that growth of larvae of the cotton bollworm *Helicoverpa armigera* (Hübner) was substantially decreased on an artificial diet containing two serine protease inhibitors but not on a diet containing one serine protease inhibitor.

### *In vivo* effect of AtSerpin1 on *A. pisum*

Cysteine proteases have been identified in several aphid species [50], [51], including *A. pisum* [33], [52], [53]. Our *in vitro* assays showed that AtSerpin1 strongly inhibits cathepsin B and L protease activities of whole *A. pisum* extracts, and when administered into an artificial diet, AtSerpin1 was toxic to *A. pisum* nymphs with 50% mortality at 637 µg ml^−1^. Researchers previously suggested that a serpin from *C. maxima* (CmPS-1) plays a role in plant defence against aphids; feeding assays established a correlation between increase in CmPS-1 within the phloem sap and the reduced ability of *M. persicae* to survive and reproduce on *C. maxima* plants [25]. However, survival of neonate *M. persicae* nymphs fed on a sucrose solution supplemented with 200 µg ml^−1^ of purified CmPS-1 was not reduced. This might be because CmPS-1 requires additional phloem proteins to form an active complex [25].

Some studies have reported deleterious effects of plant cysteine protease inhibitors on aphids fed on artificial diets. The cystatin OC-I induced moderate but significant growth inhibition on three aphid species: *A. pisum*, the cotton aphid *Aphis gossypii* Glover, and *M. persicae* [54]. Likewise, diets supplemented with OC-I (ranging from 20 to 500 µg ml^−1^) significantly reduced nymphal survival of the potato aphid *Macrosiphum euphorbiae* (Thomas) and prevented aphids from reproducing [55]. Artificial diets containing either a modified version of OC-I or the recombinant chicken egg white cystatin (CEWc) reduced the survival and growth of *M. persicae* nymphs [56]. The barley cystatin HvCPI-6 was toxic to *A. pisum* nymphs (LC_50_ = 150 µg ml^−1^) [33]. Moreover, the developmental time of *A. pisum* was significantly delayed when newborn nymphs were fed for 1 day on diet containing HvCPI-6 at 400 µg ml^−1^ and were subsequently placed on bean plants until they reached adulthood [33].

In the current study, the effect of AtSerpin1 on nymphal mortality was correlated with a significant decrease of cathepsin B and L protease activities after the nymphs fed on artificial diet containing serpin. In addition, leucine aminopeptidase activity was enhanced, suggesting a compensatory response to the inhibitory effect mediated by AtSerpin1. The overproduction of non-target proteases as a response to plant defense proteins is common in herbivorous arthropods [48], [57]. In a bioassay similar to the one described here, the ingestion of HvCPI-6 by *A. pisum* and *M. persicae* nymphs was correlated with a decrease of cathepsin B and L protease activities, and in the case of *M. persicae*, an increase of leucine aminopeptidase activity [33]. Because the artificial diet used in both studies was protein free, the results suggest that the toxicity of the serpin was not linked to disruption of food protein digestion but to the disruption of non-digestive proteases involved in other physiological processes. The cysteine protease inhibitor from rice, oryzacystatin (OC-I), not only affected the aphid *M. persicae* through digestive tract targets but also inhibited extra-digestive proteolytic activities in the hemolymph and internal organs [54]. Similar to our findings, the effects of OC-I on *M. persicae* were correlated with a reduction of a major cysteine-like protease activity in whole adult extracts [54].

### Concluding remarks

Before commercial release, GM crops must undergo an environmental risk assessment to ensure that they do not cause unacceptable detrimental effects to non-target organisms. This is especially relevant in the case of plants producing protease inhibitors, given that these inhibitors may affect many different organisms. Our *in vitro* assays showed that the serine proteases of the three non-target species tested were highly inhibited by AtSerpin1. Therefore, if GM plants producing AtSerpin1 are to be deployed in the future, the impact on non-target organisms should be taken into account and special attention should be given to the routes of exposure.

*In vivo* assays with *S. littoralis* and *A. pisum* showed very promising results for pest control by AtSerpin1. Artificial diet and plant bioassays have demonstrated that AtSerpin1 reduces the growth of *S. littoralis* larvae but does not cause mortality. For AtSerpin1 to make a meaningful contribution to plant resistance against *S. littoralis*, the efficacy of the serpin must be increased either by protein engineering [12] or by using it in combination with other protease inhibitors (see above) or with other pesticidal proteins. Interestingly, *A. pisum* nymphs incurred high mortality levels when exposed to AtSerpin1 through artificial diet. Some studies have previously shown that transgenic plants producing cysteine protease inhibitors can confer partial resistance against aphid species [33], [54], [58]. Future experiments with *A. pisum* should therefore determine whether the detrimental effect observed with an artificial diet bioassay in the current study is obtained with AtSerpin1-expressing transgenic plants.
