# Correction: Potential Use of a Serpin from *Arabidopsis* for Pest Control

**DOI:** 10.1371/annotation/099db8aa-be3a-4635-b464-dc94ba0fb069

**Published:** 2011-09-02

**Authors:** Fernando Alvarez-Alfageme, Jafar Maharramov, Laura Carrillo, Steven Vandenabeele, Dominique Vercammen, Frank Van Breusegem, Guy Smagghe

Figures 3 and 4 are swapped. The legends are correct. 

